# Extending SEQenv: a taxa-centric approach to environmental annotations of 16S rDNA sequences

**DOI:** 10.7717/peerj.3827

**Published:** 2017-10-10

**Authors:** Ali Z. Ijaz, Thomas C. Jeffries, Umer Z. Ijaz, Kelly Hamonts, Brajesh K. Singh

**Affiliations:** 1 Hawkesbury Institute for the Environment, Western Sydney University, Penrith, Australia; 2 School of Science & Health, Western Sydney University, Penrith, Australia; 3 Indigo V Expeditions, Sentosa Cove, Singapore; 4 Infrastructure and Environment Research Division, School of Engineering, University of Glasgow, Glasgow, United Kingdom

**Keywords:** Environmental, Annotations, 16S rDNA, SEQenv, Taxa centric, Biogeography

## Abstract

Understanding how the environment selects a given taxon and the diversity patterns that emerge as a result of environmental filtering can dramatically improve our ability to analyse any environment in depth as well as advancing our knowledge on how the response of different taxa can impact each other and ecosystem functions. Most of the work investigating microbial biogeography has been site-specific, and logical environmental factors, rather than geographical location, may be more influential on microbial diversity. SEQenv, a novel pipeline aiming to provide environmental annotations of sequences emerged to provide a consistent description of the environmental niches using the ENVO ontology. While the pipeline provides a list of environmental terms on the basis of sample datasets and, therefore, the annotations obtained are at the dataset level, it lacks a taxa centric approach to environmental annotation. The work here describes an extension developed to enhance the SEQenv pipeline, which provided the means to directly generate environmental annotations for taxa under different contexts. 16S rDNA amplicon datasets belonging to distinct biomes were selected to illustrate the applicability of the extended SEQenv pipeline. A literature survey of the results demonstrates the immense importance of sequence level environmental annotations by illustrating the distribution of both taxa across environments as well as the various environmental sources of a specific taxon. Significantly enhancing the SEQenv pipeline in the process, this information would be valuable to any biologist seeking to understand the various taxa present in the habitat and the environment they originated from, enabling a more thorough analysis of which lineages are abundant in certain habitats and the recovery of patterns in taxon distribution across different habitats and environmental gradients.

## Introduction

Microbial communities are genetically diverse, and occupy every known habitat where they participate in driving nutrient cycles and form the basis of food webs. The niche of these organisms however, is influenced by environmental characteristics especially in the context of the Baas-Becking hypothesis (Baas-Becking, 1934), which states that, “everything is everywhere but the environment selects (De Wit & Bouvier, 2006).” This determines relative abundance and patterns in diversity of microbial communities.

There is an increasing interest in comprehensive description of environmental context and experimental methods used for sequencing data, without which such data sets would be of less value for comparative studies or discovering linkages between genetic potential and the diversity and abundances of organisms (Field et al., 2008). Furthermore, a full understanding of the role of environmental selection of microbial diversity can only be realised if associated metadata related to geographical or environmental information can be exploited.

To that end, various formal specifications and guidelines have been developed to facilitate curation of metadata in a standardised format such as the minimum information about any sequence specifications (Yilmaz et al., 2011) by the Genomic Standards Consortium (Field et al., 2011). Furthermore, sequence data submission to many public databases including GenBank (Benson et al., 2012) and INSDC (Nakamura, Cochrane & Karsch-Mizrachi, 2013) as well as online bioinformatics tools like MG-RAST (Aziz et al., 2008) have specific metadata fields for storing contextual information concerning the sequences. Moreover, large scale projects such as the Earth Microbiome Project (Gilbert, Jansson & Knight, 2014), which aim to develop a global catalogue of microbial diversity, store contextual metadata information as well.

Before applicable environmental annotation can be performed for sequences, a precise and consistent environmental description for the origins of these sequences and the samples they came from, is needed. The Environmental Ontology, or ENVO Ontology, provides a structured, controlled vocabulary in a hierarchical list of descriptors, which can then be used to organize environmental data in a coherent and unambiguous manner (Buttigieg et al., 2013). In essence, the ontology provides a list of standardized environment descriptors that can be used to properly explain the environment or habitat as well as its noticeable features and has been adopted by MG-RAST (Aziz et al., 2008), the iMicrobe project (Buttigieg et al., 2016) and Earth Microbiome Project (Gilbert, Jansson & Knight, 2014).

The NCBI-NT database provides a wealth of information with respect to environmental metadata. Sequences submitted to the database may contain a GenBank (Benson et al., 2012) metadata field known as *isolation source*, which provides the environment source from where the organism was extracted from (National Center for Biotechnology Information, 2011). This can then be exploited to label sequences with the necessary environmental annotation and can enable characterization of any ecological project with respect to environmental terms using the ENVO ontology.

SEQenv (Sinclair et al., 2016) is a new, cutting edge pipeline, which can generate environmental information for sequences, primarily using the isolation source metadata field from NCBI-NT. The pipeline begins by retrieving highly similar sequences from the NCBI-NT database using the BLASTN algorithm (Altschul et al., 1990). From the hits that match against the query sequences, text fields carrying environmental information such as isolation sources found in the metadata are extracted. Given that isolation sources are in the form of short English sentences, this information is converted into the nearest ENVO ontology terms (Buttigieg et al., 2013). The pipeline is uniquely placed to derive environmental annotations for sequences as so far no automated bioinformatics pipeline exist for this purpose. Lastly, the pipeline can be used for both nucleotides and protein sequences (Sinclair et al., 2016).

However, SEQenv is only able to generate a list of environmental terms on the basis of sample datasets and lacks a taxa centric approach to environmental annotations. Hence, sequence level environmental information is not provided. Such information is critical to identify niches for a particular taxon and the potential role of key taxa in driving ecosystem functions, and therefore necessitated an enhancement to the pipeline to provide a more contextual, taxa oriented view of the environmental annotations.

This study aimed to address these deficiencies by developing a taxa-centric extension to SEQenv pipeline, which consisted of two parts, each providing environmental annotations under different context, with first part providing taxon abundance on a per term basis while the second part lists environmental term abundance under a per taxon context. A separately developed program that required the use of the original SEQenv pipeline, this enabled two different methods of viewing environmental annotations, which significantly augments the analysis capability of the pipeline. The extended pipeline was integrated with the TaxaSE system (Ijaz, 2017b), available at (Ijaz, 2017a), which is a per-sequence taxonomic annotation system that utilizes Shannon entropy to quantitatively determine sequence similarity as opposed to percentage identity, providing high-resolution taxa level information.

Two amplicon datasets belonging to distinct biomes were selected in order to determine the applicability of both the SEQenv pipeline and the newly developed extension, towards environmental annotation of different habitats and to determine if the extension provided a environmentally supported and correct view of taxa distribution. Lastly, results were visually illustrated for improved readability.

## Materials & Methods

### Integration with the TaxaSE System

As the TaxaSE system (Ijaz, 2017b) followed an OTU independent approach, new tools were developed to select unique sequences from taxonomic annotation results that can then be given to SEQenv pipeline for environmental tagging. The approach followed is as follows:

(1) From the distinct taxonomic annotation results of TaxaSE system, a collection of sequences was selected on the basis of a genus level threshold.
(2) Relative abundances of the taxa were generated for every annotation result.
(3) Sequences belonging to every taxon were randomly selected. The number of sequences selected was directly proportional to the relative abundance in the collection of the sequences. For example, a genus with higher relative abundance had more sequences selected from it compared to a genus with lower relative abundance.
(4) The random selection ensured that a wide variety of sequences were used for analysis and were representative of the sample diversity.

**Figure 1:**
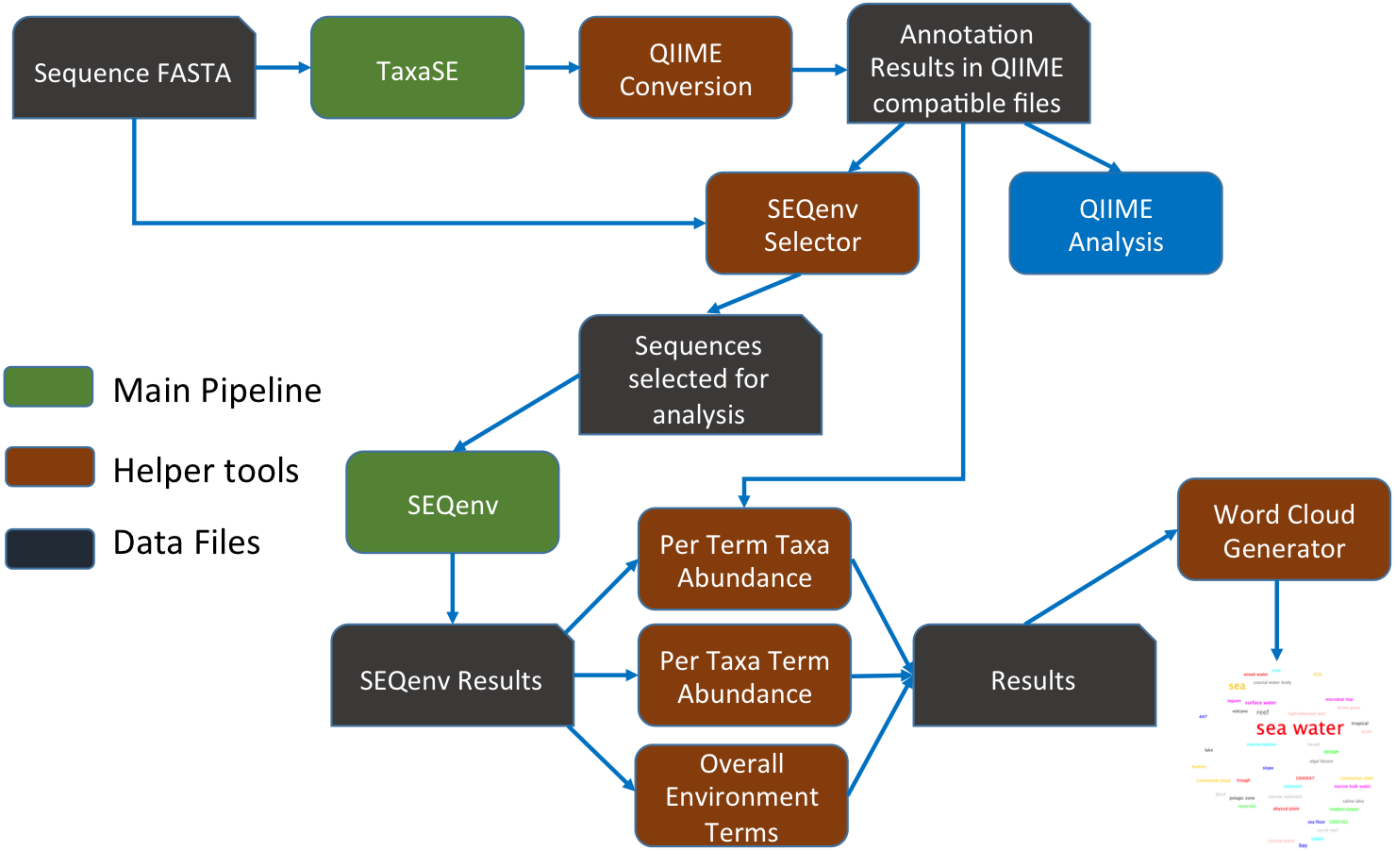
Integration and enhancement of SEQenv system, with pipelines shown in green, helper tools in brown and data files in black.

The integration of the SEQenv pipeline as well as the extension developed is illustrated in Fig. 1.

### Per environmental term taxa abundance

A taxon abundance or contribution to each environment terms provides more detailed information and can help understand which sequences may be more important in contributing to a particular environment. Building upon the current version of SEQenv (Sinclair et al., 2016) by extracting taxa abundance for a given environmental term, allowed for the opportunity for detailed analysis of the partitioning of diversity across habitats within the context of the samples being analysed.

SEQenv results consisted of a global matrix between sequences and their associated environment terms. Given that taxonomic annotation information was present for these sequences via the TaxaSE system, a per environment term taxa abundance result was therefore generated. In essence, for every environment term, a list of ranked taxa was produced. The ranking of these taxa was dependent upon how much they contributed to the specific term.

(1) For every environment term, select the sequences that contributed towards it. These were taken from SEQenv results. As a sequence may have multiple terms associated with it, hence the contribution value of the specific term is used. For example, a sequence may have 50% soil and 50% forest soil as the environmental terms, therefore for “soil” environmental term, the contributing value 0.5, after conversion to decimal, is used downstream.
(2) For each of these sequences, the associated taxonomic annotation information was recovered from TaxaSE results.
(3) If one or more sequences belonged to the same taxonomic annotation, the contribution by each sequence was added together. Hence, the abundance denoted how much each taxon contributed towards the specific environmental term. This was performed for all sequences.
(4) Taxonomic annotations were then ranked according to how much they contributed to the environment term.

### Per taxon environmental term abundance

Relating environmental information to sequences in a direct fashion will improve our understanding of how taxa are distributed across various environments and would be a valuable asset for any biologist aiming to understand the natural habitats and niche specificity of these microbes. Hence, using the same global matrix acquired from SEQenv, a list of sequences and the environments they belong to was created in the following manner:

(1) A list of environmental terms for every sequence was generated from SEQenv results. Similar to Per Term Taxa Abundance, a sequence may have multiple environmental terms associated with it. However, here the terms were grouped on the basis of taxonomic annotation.
(2) Taxonomic annotation information from TaxaSE system results was recovered and the sequences were assigned the corresponding taxonomy.
(3) If one or more sequences had the same taxonomy, the contribution by each environmental term was added together. Therefore, abundance in this context denoted how much each environmental term was the isolation source for the specific taxon. For example, if two sequences belonging to the same taxon had 0.3 and 0.2 for “soil” environmental term respectively, then the aggregate value of 0.5 is used downstream.
(4) Environmental terms were then ranked according to how much they contributed to the taxa.

### Datasets

To illustrate the effectiveness of the extended SEQenv pipeline, datasets belonging to distinct and diverse biomes were selected. These datasets included soil, rhizosphere and plant microbiome from sugarcane (*Saccharum* spp.) sequenced by Dr. Kelly Hamonts at Hawkesbury Institute for the Environment, Western Sydney University, Australia (Supplemental Information 1) and samples from two distinct marine sub habitats (Jeffries et al., 2015). The number of sequences selected from these datasets is given in Table 1. SEQenv version 1.1.0 was run with default parameters using BLASTN (Supplemental Information 1).

**Table 1:** Datasets selected for analysis with enhanced SEQenv pipeline.

Habitat	Sub habitat	Total number of sequences
Sugarcane	Rhizosphere	3,000
	Soil	3,000
	Stem	3,000
	Root	3,000
Marine	Coral Atoll	1,500
	Southern Ocean	1,500

### Analysis approaches

In the context of this study, analysis of the datasets were divided into three sections:

• *Per Habitat Environmental Terms*: This represented the environmental terms as generated by the main SEQenv pipeline.
• *Per Environmental Term Taxa Abundance*: This represented the taxonomic abundance as generated by the first part of the new extension to the SEQenv pipeline. Furthermore, SEQenv results for each sub-habitat from the aforementioned datasets were aggregated and Per Term Taxa Abundance results were then generated from the resultant information.
• *Per Taxon Environmental Term Abundance*: This represented the environmental terms abundance on a per taxon basis, as generated by the second part of the new extension to SEQenv pipeline. Similar to per environmental term taxa abundance, SEQenv results for each sub-habitat were aggregated.

## Results

### Per habitat environmental terms

#### Sugarcane dataset

**Figure 2:**
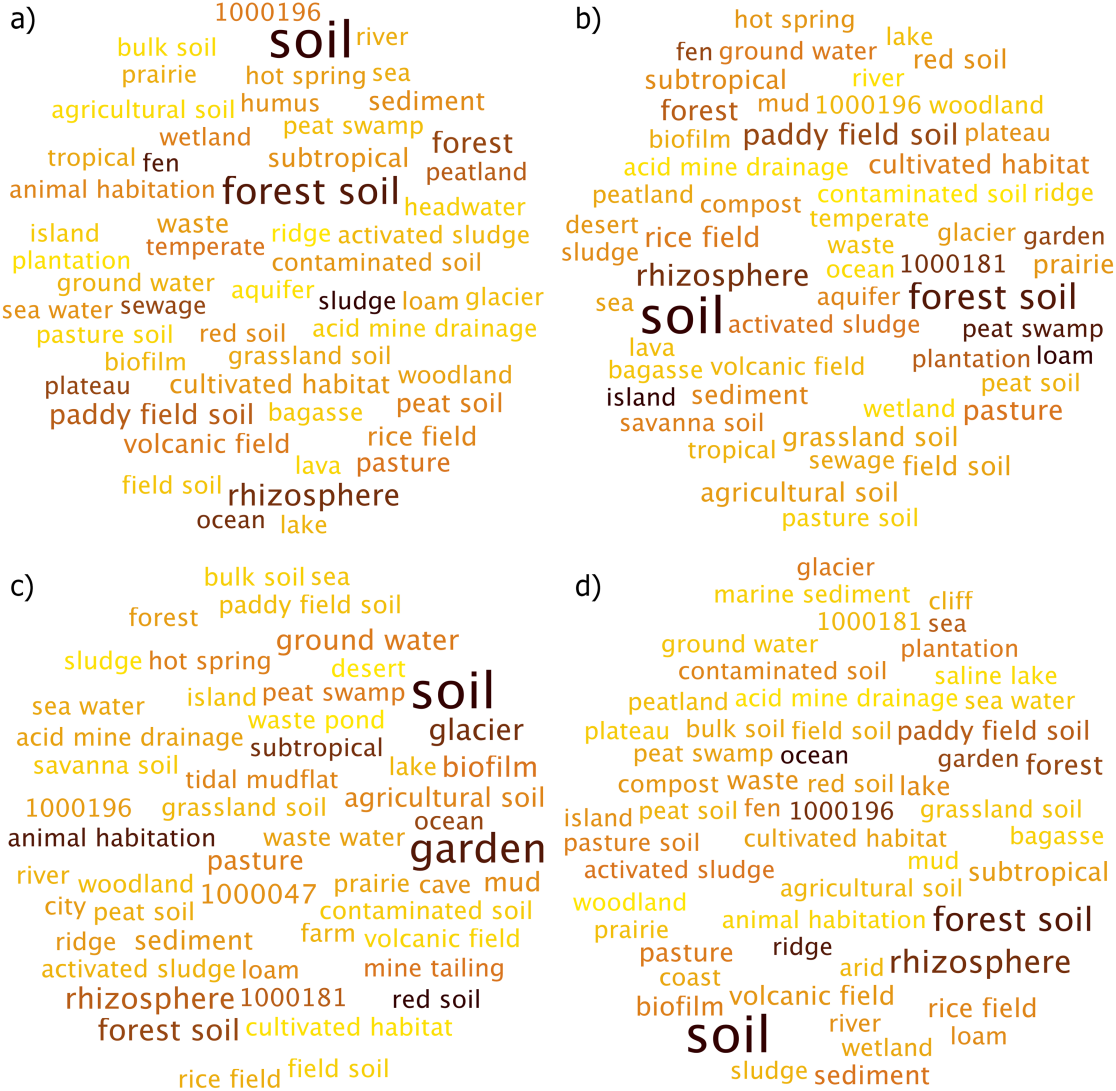
Environmental terms generated for the sub-habitats (A) rhizosphere (B) soil (C) stem and (D) root. More abundant terms are highlighted in darker color and larger font.

The environmental terms for the sugarcane dataset are illustrated in Fig. 2. Samples belonging to rhizosphere showed the environmental term “soil” as being the most prevalent (Fig. 2A). Other similar terms were also observed, such as “rhizosphere”, “forest soil”, “prairie” and “agricultural soil”. Of importance was the occurrence of the environmental terms such as “activated sludge”, “garden” and “contaminated soil” as more taxa with these metadata were prevalent in these datasets. Soil samples illustrated a similar collection of environment terms, with the “soil” term being the most significant environmental term observed (Fig. 2B). However, “forest soil” was observed relatively strongly here compared to rhizosphere results.

For the stem samples, the environmental term “garden” was strongly observed compared to other terms, with exception of “soil” (Fig. 2C). Other important terms included “forest soil”, “biofilm” and “garden soil”. Lastly, the root samples showed similarity to both soil and rhizosphere samples, where the environmental term “soil” was the most observed term (Fig. 2D). While the plots show similar list of environmental terms, with exception of the few most strongly observed terms, the ranking of the terms themselves vary across these habitats.

Across these different samples, SEQenv was unable to generate environmental terms for some ENVO IDs. This included 1000196, which stood for “coniferous forest biome”, 446, which was “terrestrial biome” and lastly 447, which was “marine biome”, 1000181, which was “mangrove biome”, 428, which was simply “biome” and 2030, which was “aquatic biome”.

Overall, the environmental term “soil” was prevalent across all sub-habitats, however other terms were ranked differently. Stem sub-habitat was more unique compared to soil, rhizsophere and root. This is also illustrated in Table 2, where the top 10 environmental terms are ranked according to their abundances for each sub-habitat, with the differences between sub-habitats highlighted in bold.

**Table 2:** Top 10 environmental terms observed in sub-habitats from sugarcane dataset, sorted in a descending order of abundance and unique terms highlighted in bold.

	Sub-habitats			
Rank	Soil	Rhz	Root	Stem
1	soil	soil	soil	soil
2	forest soil	forest soil	forest soil	**garden**
3	rhizosphere	rhizosphere	rhizosphere	**glacier**
4	paddy field soil	forest	forest	**ground water**
5	rice field	paddy field soil	paddy field soil	forest soil
6	forest	pasture	sediment	biofilm
7	pasture	volcanic field	rice field	rhizosphere
8	cultivated habitat	rice field	pasture	pasture
9	sediment	subtropical	biofilm	agricultural soil
10	subtropical	cultivated habitat	cultivated habitat	mud

#### Marine dataset

**Figure 3:**
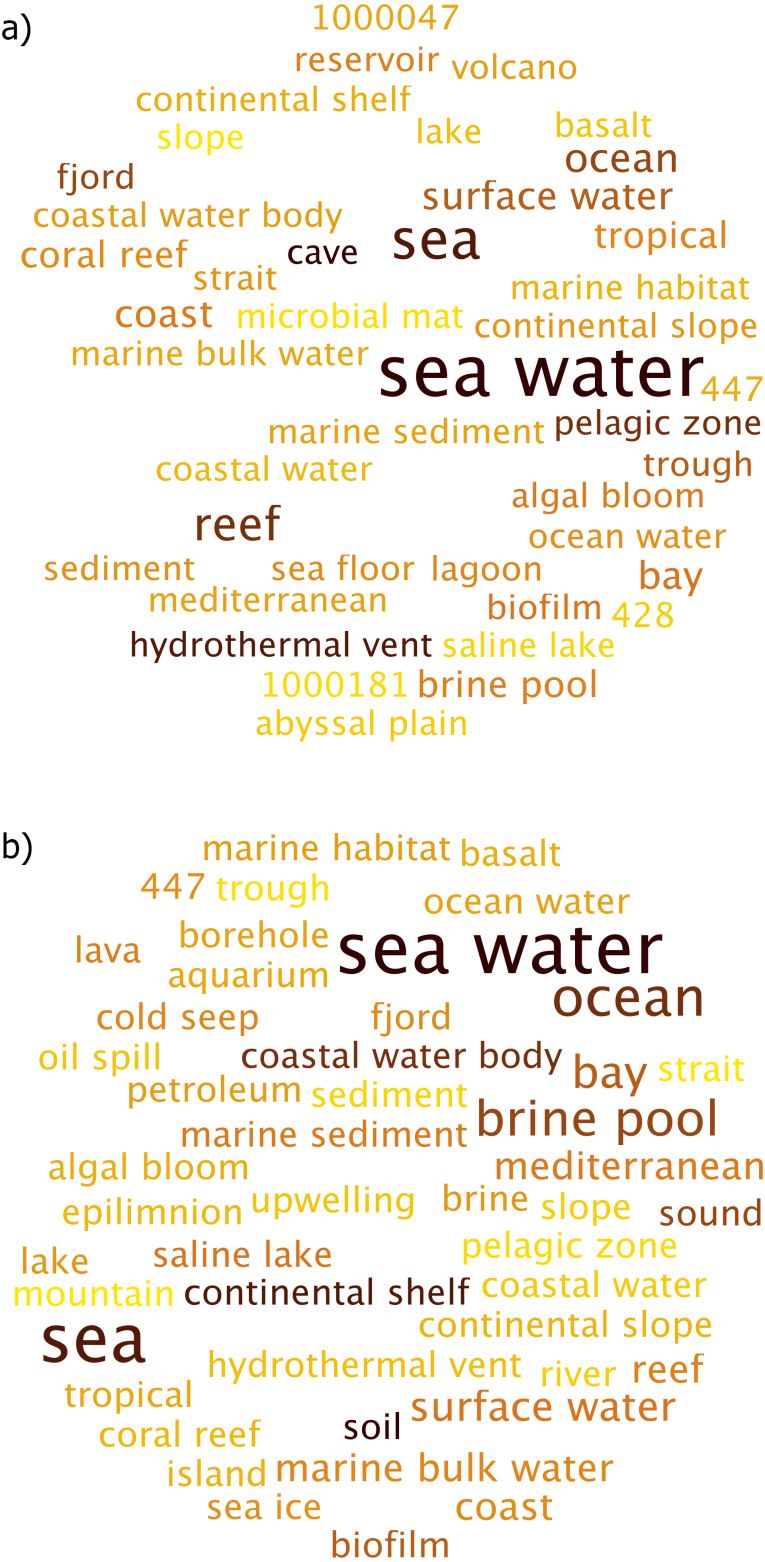
Environmental terms for the marine sub-habitats (A) Coral Atoll and (B) Southern Ocean. More abundant terms are highlighted in darker color and larger font.

For the coral atoll marine samples, the environmental term “sea water” was the most observed term, with “sea” coming up after that (Fig. 3A). A few other terms of importance include “bay”, “coral reef”, “ocean” and “sediment”. Southern ocean samples also showed a similar list of environmental term (Fig. 3B). Here as well “sea water” and “sea” terms were the most observed; however, “brine pool” was relatively strongly observed here compared to samples from coral atoll.

Similar to samples from sugarcane dataset, SEQenv was unable to determine the environmental term for the IDs 428, which was “biome”, 447, which was “marine biome” and 1000047, which was “mediterranean sea biome”.

While both marine samples showed a similar list of environment terms, these differed in the ranking of the terms themselves, which is illustrated in Table 3. Here, the ranking of top level environmental terms were the same for both coral atoll and southern ocean samples, however differences were observed in the lower ranked terms where “coral reef” was observed for coral atoll samples while southern ocean had environmental terms like “Mediterranean” and “marine bulk water”, which were absent in coral atoll samples.

### Per environmental term taxa abundance

#### Sugarcane dataset

**Figure 4:**
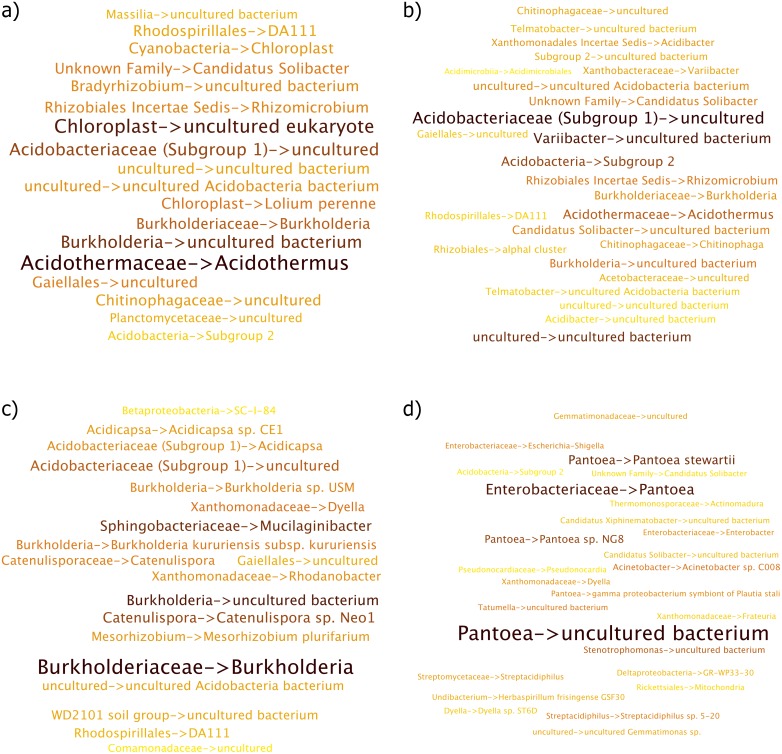
Per Term Taxa Abundance for the environmental terms (A) soil (B) forest soil (C) rhizosphere and (D) garden. More abundant taxa are highlighted in darker colors and larger font.

While the environment terms “Soil” and “Forest Soil” were similar, the sequences that contribute to these terms differed (Fig. 4A and 4B respectively). This was quite apparent in the differences between both word clouds where the most abundant taxon for “soil” term included *Acidothermus* and *Chloroplast*, which is potentially a misclassified Cyanobacteria while *Variibacter* and *Acidobacteriaceae* were more strongly related to the “forest soil” term.

**Table 3:** Top 10 environmental terms observed in sub-habitats from marine dataset, sorted in descending order and unique terms highlighted in bold.

	Sub-habitats	
Rank	Coral Atoll	Southern Ocean
1	sea water	sea water
2	sea	sea
3	reef	ocean
4	ocean	brine pool
5	surface water	bay
6	bay	surface water
7	coast	**mediterranean**
8	brine pool	reef
9	tropical	**marine bulk water**
10	**coral reef**	coast

“Rhizosphere” environmental term had *Burkholderia* as being the most abundant taxon while *Acidothermus* was almost non-existent in this case as shown in Fig. 4C. *Burkholderia* was followed by *Catenulispora sp. Neo1*, *Acidobacteriaceae (Subgroup 1)* and *Dyella*.

The “garden” environmental term had distinct taxa, which were not observed in other environmental terms (Fig. 4D), with the members of *Pantoea* genus making up the collection of taxa.

**Figure 5:**
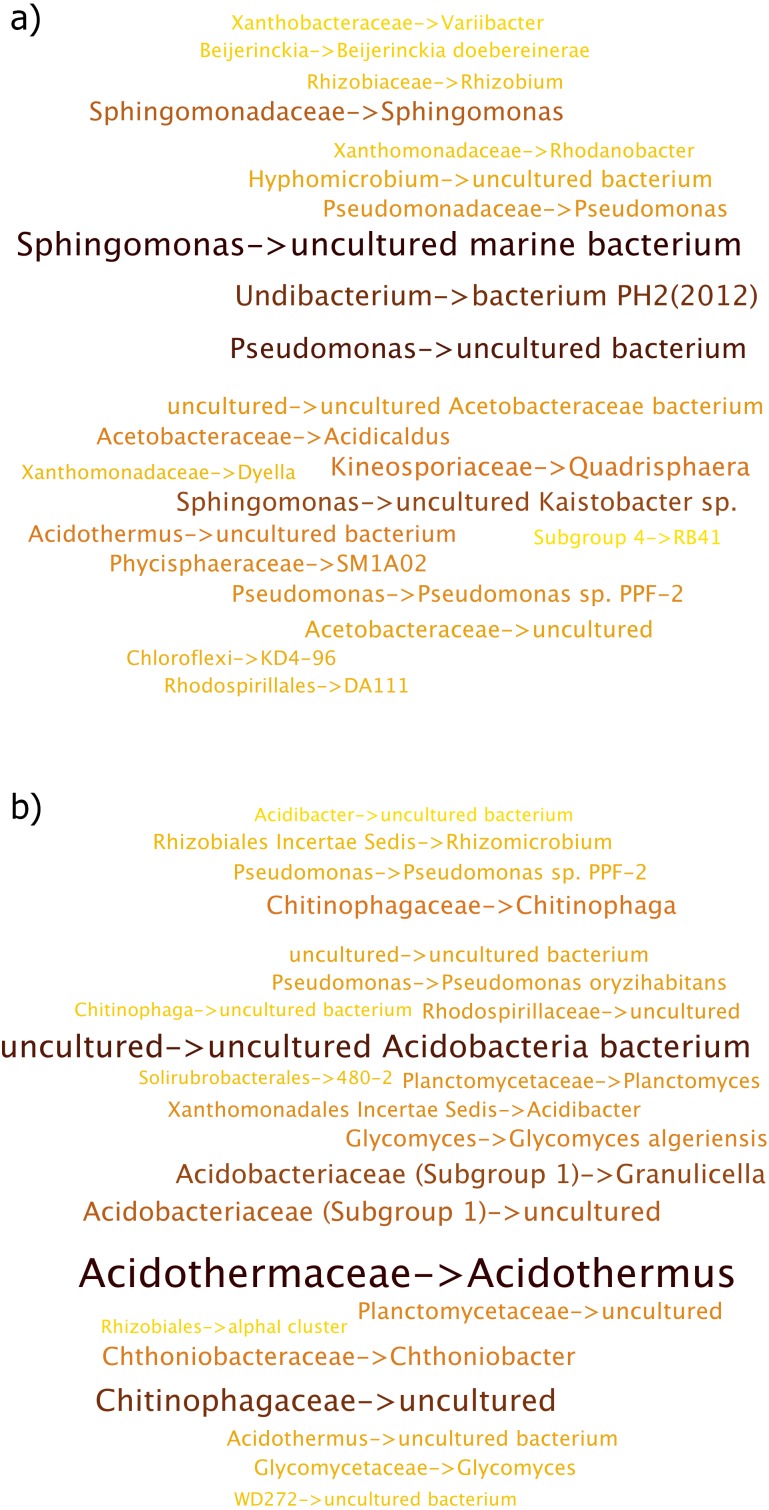
Per Term Taxa Abundance for environmental terms (A) contaminated soil and (B) waste. More abundant taxa are highlighted in darker color and larger font.

The “Contaminated soil” term is an example of significantly different collection of taxa (Fig. 5A). While not listed in the top 10 environmental terms for the sugarcane dataset, it and “waste” environmental term consists of important collection of taxa. Here, *Sphingomonas*, *Pseudomonas* and *Undibacterium* were more abundant, in that order.

For the “waste” environmental term, *Acidothermus* was the most observed taxon, followed by *Acidobacteriaceae (Subgroup 1)* (Fig. 5B). Additionally, members of *Chitinophagaceae* family were also seen under this environmental term.

#### Marine dataset

**Figure 6:**
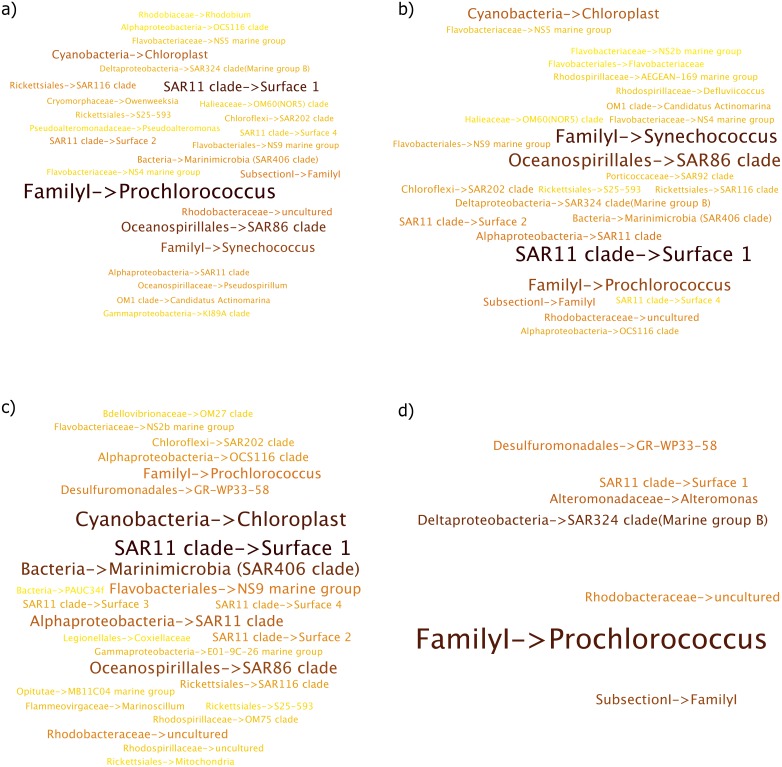
Per Term Taxa Abundance for the environmental terms (A) sea water (B) sea (C) ocean and (D) brine pool. More abundant taxa are highlighted in darker color and larger font.

*Prochlorococcus* dominated the taxa abundance for the environmental term “sea water” (Fig. 6A). The other taxa such as *SAR11 clade* and *SAR86 clade* were also observed, though at a lower abundances. On the other hand, while similar taxa was observed for the environmental term “sea”, the relative abundance of these taxa were significantly different (Fig. 6B). *SAR11 clade* and *Synechococcus* became more abundant, while *Prochlorococcus* was observed to be far less prominent than what was observed for the environmental term “sea water”.

“Ocean” environmental term showed *Chloroplast* becoming more abundant compared to per term taxa abundance for “seawater” and “sea” environmental terms (Fig. 6C). Similar behaviour was seen for *Marinimicrobia (SAR406 clade)* as well, which was very low in abundance in the aforementioned environmental terms.

*SAR86 Clade* and *Prochlorococcus* jointly dominated the “Brine Pool” environment term (Fig. 6D). Furthermore, a few taxa such as *SAR324 clade (Marine group B)* and *Alteromonas* were also observed, although at a very low abundance. Species diversity was observed to be quite low in this case as only a few taxa contributed to this environment term. Overall, most of the taxa belonged to *Proteobacteria* and *Cyanobacteria* phyla.

### Per taxon environmental term abundance

#### Sugarcane dataset

**Figure 7:**
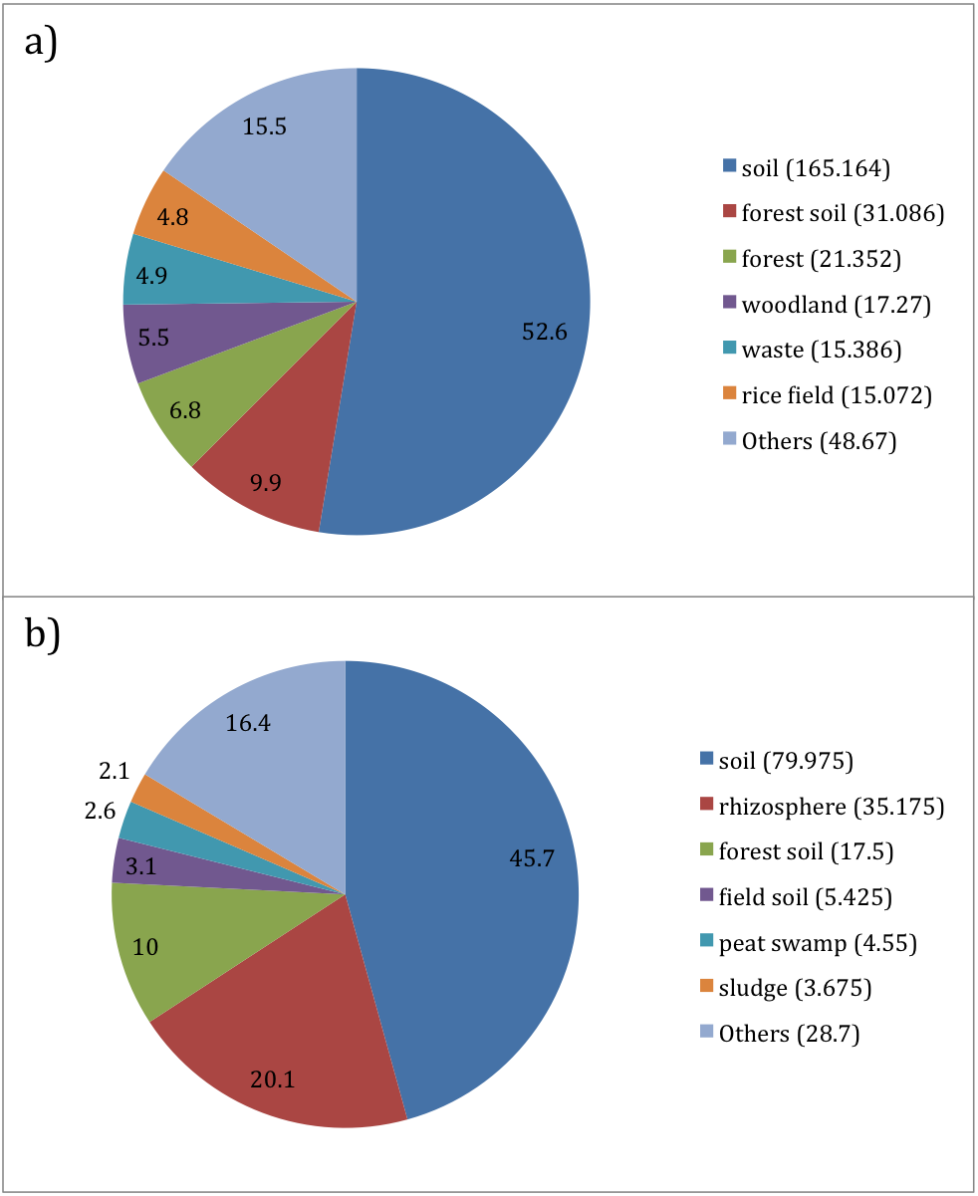
Per Taxon Term Abundance for (A) Acidothermus (314 sequences) and (B) Bulkholderia (175 sequences). Top 6 environmental terms are illustrated with the pie chart. Values in the legend denote approximate number of sequences contributing to the environmental term.

Per taxon environmental term relative abundance for *Acidothermus* and *Burkholderia* is illustrated in Fig. 7. While the environmental term “soil” dominated the list of terms for both genera, 52.6% for *Acidothermus* and 45.7% for *Burkholderia*, differences were observed for the lower ranked terms, where “Forest soil” was the second most observed term for *Acidothermus* at 9.9% (Fig. 7A), however for *Bulkholderia* the term “rhizosphere” was observed higher than “forest soil”, accounting for a significant portion of the environment terms at 20% and “forest soil” term accounting for 10% here, similar to *Acidothermus*.

The terms “woodland”, “waste” and “rice field” were ranked higher for *Acidothermus* (Fig. 7A) as well, at 5.5%, 4.9% and 4.8% respectively. For *Bulkholderia*, the “waste” term was not in the top 6 environmental terms (Fig. 7B), and furthermore the terms “woodland” and “rice field” were not observed for this genus.

“Field soil”, “peat swamp” and “sludge” environmental terms were observed for *Bulkholderia* at 3.1%, 2.6% and 2.1% respectively, however they were absent from the collection of top 6 terms for *Acidothermus*. Lastly, the remaining collection of environmental terms came at 15.5% for *Acidothermus* and 16.4% for *Bulkholderia*.

Overall, distinct differences were observed between both genera. For *Acidothermus*, with exception of the most abundant “soil” term, others gradually decreased in how much they accounted for in the list of environmental terms. However *Bulkholderia* showed the gradual decrease after the third ranked “forest soil” term.

#### Marine dataset

**Figure 8:**
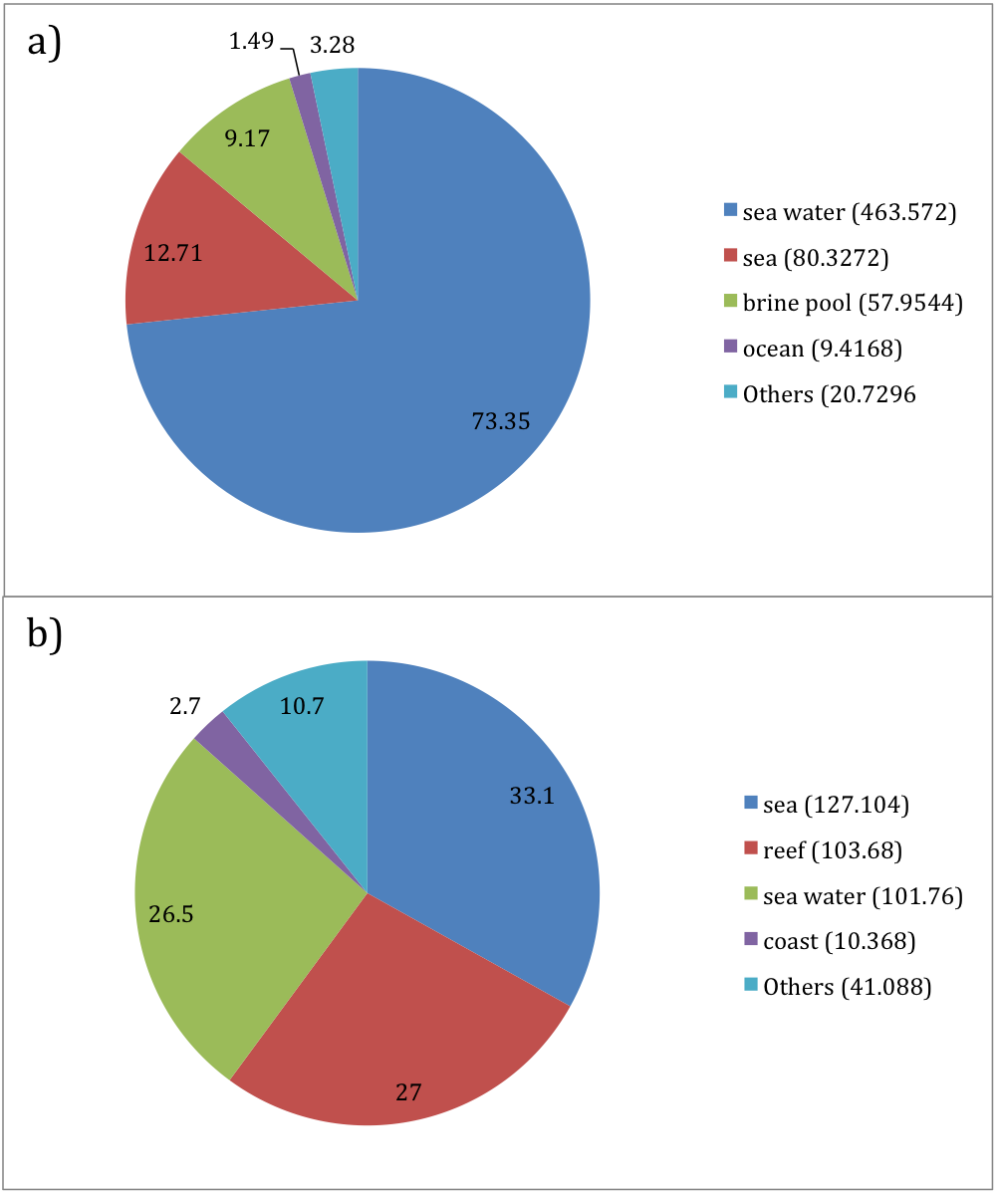
Per Taxon Term Abundance for (A) Prochlorococcus (632 sequences) and (B) Synechococcus (384 sequences). Top 4 environmental terms are illustrated with the pie chart. Values in the legend denote approximate number of sequences contributing to the environmental term.

The per taxon term abundance pie charts for the genus *Prochlorococcus* and *Synechococcus* are illustrated in Fig. 8. For *Prochlorococcus*, the environmental terms “sea water” was the most observed term, accounting for 73.35% of environmental terms, an overall majority (Fig. 8A), which came down to third rank for *Synechococcus*, at 26.5% (Fig. 8B).

Furthermore, “reef” was observed strongly for *Synechococcus* at 27%, however the term was absent in the top 4 list for *Prochlorococcus*. Furthermore, “ocean” term was present for *Prochlorococcus* at 1.49%. Other differences included the term “brine pool” at 9.17% for *Prochlorococcus*, although it was absent for *Synechococcus*. Lastly, “coast” environmental term was observed only for *Synechococcus* at 2.7%.

Overall, environmental term distribution was different between both genera. A single “Sea water” term dominated *Prochlorococcus* list of environmental terms while *Synechococcus* saw three terms accounting for most of the environmental terms observed, on an almost equal level and where “reef” term was distinctly observed for *Synechococcus*.

## Discussion

Sequence annotation can now be enhanced with environmental data, by way of exploiting information available in associated metadata in databases such as NCBI-NT. This can in turn provide a more in-depth view into the microbial community and a more effective approach towards analysis for many ecological projects.

The analysis of the various habitats illustrates the effectiveness of the new extension to SEQenv. Significant patterns emerge where distinct taxa were strongly observed on the basis of the environment origin. By effectively linking observed taxa to environmental terms, the system produces an ecologically important perspective into the analysis of 16S rRNA sequences and enables a more thorough approach to environmental annotation of sequences, aiding in interpretation of taxonomic annotation.

Cases were found where the SEQenv pipeline (Sinclair et al., 2016) was unable to resolve the environmental term at a deeper level, such as for the environmental terms “soil” and “sea”. Given that “soil” term exists at a higher level than other terms such as “forest soil” in ENVO ontology (Buttigieg et al., 2013), it is more likely that the isolation sources for these sequences were not detailed enough to determine the precise environment they were isolated from. SEQenv selected a higher level of environment term instead, as the metadata could not provide more specific details about the environment. Additionally, some ENVO IDs could not be resolved to the proper environmental terms, which may be a limitation of the tool as some of these IDs were similar across various sub-habitats.

### Per habitat environmental terms

#### Sugarcane dataset

While the results for the root sub-habitat were similar to soil and rhizosphere, differences were observed for the presence of environmental terms such as “sediment” and “biofilm”, which were ranked higher. This might be because of the taxa that belonged to these terms was more abundant in the root habitat due to plant-soil close association (Garbeva, Veen & Elsas, 2004). Furthermore, biofilms play an important role in plant-microbial interactions in the rhizsophere (Danhorn & Fuqua, 2007). Additionally “forest soil” environmental term was relatively more prominent for soil samples compared to rhizosphere results and this might be due to difference in abundance of taxa that are more prevalent in forest soils, which are located further away from the phytobiome system (Garbeva, Veen & Elsas, 2004).

The differences between stem samples and others, which was driven by terms such as “garden”, “glacier water” and “ground water” can be explained by the these terms being driven by taxa unique to the stem habitat and likely to be endophyte in nature. These taxa live within the plant biomass in a symbiotic relationship (Gouda et al., 2016) and therefore observed in the samples taken from the stem. This may be a result of comparatively lower number of sequences from these habitats exist in the database. Nonetheless, given that SEQenv (Sinclair et al., 2016) acquires isolation sources based on the sequences in the dataset, the differences in species found in the stem samples compared to other samples led to strong difference in environmental based tagging. Furthermore, as the stem samples were taken from the stem of sugarcane plants, the ranking of environmental terms in this case are a good representative of the type of the environment the microbial sequences came from. This highlights the value of SEQenv in discriminating between habitats.

#### Marine dataset

While most of the environmental terms observed for the two different marine based sub-habitats were similar, the ranking of the terms themselves were different and some environmental terms were uniquely observed such as “coral reef” environmental term for coral atoll sample, due to differences in the environment between these two sub-habitats and the variation in taxa abundance that comes with it (Jeffries et al., 2015). Some microbial communities in coral reef systems exist in a symbiotic relationship with coral polyps, playing a role in nutrient cycling as well as assisting in disease resistance for these organisms (Garren & Azam, 2012). Therefore taxa belonging to this environment are more likely to be observed for coral atoll samples. “Marine bulk water” was uniquely observed for southern ocean samples while being absent for coral atoll samples, due to the environmental characteristic of the ocean waters and the taxa that are prevalent in it.

For all the datasets used for analysis, it was apparent that SEQenv was able to determine intra-habitat differences and patterns even at environmental term level of information.

### Per environmental term taxa abundance

As seen in the word clouds for the habitats, certain environmental terms were more strongly observed compared to others. Underpinning this pattern is the taxa abundance, which contributed to their ranking. The Per Environmental Term Taxa Abundance approach was able to provide a more taxa centric explanation of these patterns, which could not be explained solely by SEQenv (Sinclair et al., 2016).

Per Environmental Term Taxa Abundance showed distinct patterns of taxa abundances across various environmental terms. Taxa more prevalent in one term were less abundant in another. Certain taxa had low abundances; however, depending on the environmental factors, these taxa can become more abundant if the conditions are beneficial towards their growth.

#### Sugarcane dataset

The difference in the abundance of taxa between “rhizosphere” and “soil” terms illustrate that while some taxa were common across different environment, the abundances observed were different.

*Acidothermus*, which was strongly observed in the “soil” environment term, is a thermophilic, acidophilic, cellulolytic bacterium, prevalent in acidic environments (Mohagheghi et al., 1986), while *Acidobacteriaceae* as observed more in the “forest soil” environment term, is a family of *Acidobacteria* which are ubiquitous in soil environment (Quaiser et al., 2003).

The “garden” environment term was significantly different from other terms in the case of the sequences that contributed to it where “*Pantoea*” was the most abundant taxa observed. It is well known that *Pantoea Spp.* lives in many plant tissue both as commensal and in some cases as pathogens (Pataky et al., 2000).

Members of the *Sphingomonas* genus were observed for the environmental term “contaminated soil” and bacteria belonging to this genus is well known to have the ability to degrade chemicals in contaminated soil as it is one of the best known genus for biodegradation of chemical contaminants (Alvarez et al., 2012; Schmidt et al., 1992; Ye et al., 1995). The most prevalent species of *Sphingomonas* was observed to be an “uncultured marine bacterium”. The presence of this bacterium here may be due to this taxa being prevalent in both contaminated soil and marine habitats.

Lastly, while taxa that contributed to the terms such as “contaminated soil” and “waste” were not as abundant as the aforementioned terms like “soil”, “forest soil” or “garden”, they were nonetheless very important as they provided taxa abundances under a specific environmental context. Therefore, for studies that may aim towards a specific goal in mind, such as bioremediation, this may help in targeting sequences that come from relevant environments.

#### Marine dataset

Overall, Marine habitats showed an interesting collection of taxa that come from a variety of marine environments. Similar to the sugarcane dataset, while the list of sequences contributing to each environment may seem similar at first, there were exceptions where unique sequences were observed to be more abundant in specific environments. Furthermore, the ranking itself varied across every environmental term. Additionally, similar to the differences observed for “soil” and “forest soil” environmental terms in the sugarcane dataset, “sea” and “seawater” exhibited the same pattern with respect to taxa observed.

*Prochlorococcus*, which was observed in multiple environmental terms such as “sea water”, “sea” and “ocean” in different abundances, is a very small marine cyanobacteria, which is one of the most abundant photosynthetic organism on the planet (Partensky, Hess & Vaulot, 1999), while bacteria belonging to *SAR11 clade* are accountable for methane dissolved in the oceans (Carini et al., 2014) and are dominant marine heterotrophs. They are cosmopolitan and abundant across marine habitats, particularly *SAR11* (Brown et al., 2012), which is a highly abundant marine bacterium and was present for most environment terms at different abundances.

*Synechococcus* is a unicellular cyanobacteria that is prevalent in the marine environment and has been shown to dominate in this system (Jeffries et al., 2015). It was present for the environmental terms “sea” and “sea water”, while being absent in top 10 ranked list of taxa for the “ocean” term. *SAR86 Clade*, members of which are aerobic chemoheterotroph (Dupont et al., 2012), and the aforementioned *Prochlorococcus* jointly dominated the “Brine Pool” environment term.

The per environment term taxa abundance provided a more concise and relevant view of the environmental annotations. Linking sequences to environmental terms in such a manner would be more suitable than a list of environmental terms that SEQenv provides. This enhancement significantly improved the analysis capability of SEQenv system and provided a novel approach to contextual, taxa based environmental annotation, which was originally not present in the SEQenv pipeline. Furthermore, the integration developed here enabled a more thorough approach towards 16S rRNA sequence analysis and offers a single pipeline for both taxonomic and environmental annotation of sequences.

### Per taxon environmental term abundance

Following up on per term taxa abundance, similar patterns were observed for per taxon term abundance where certain environmental terms were dominant for specific genus. The per taxon environmental term abundance provided a taxa centric approach toward environmental annotations and listed the many habitats under which a taxon may be found.

Terms such as “sea” and “soil” are more prevalent due to the limitations associated with the SEQenv pipeline or the metadata for these sequences were not specific enough with respect to the environments they were isolated from.

#### Sugarcane dataset

In accordance with per term taxa abundance result for “soil” environment term, “soil” dominated the list of terms for *Acidothermus*, which is a thermophilic and acidophilic microbe that is found in acidic environment (Mohagheghi et al., 1986). Other terms such as “forest soil” or “woodland” point towards these environments being favourable to its growth, as it has been observed in samples collected from forest environment (Kim et al., 2015; Meng et al., 2013).

*Burkholderia* occupies a variety of environmental niches (Compant et al., 2008) including soil (Janssen, 2006) and some strains of this genus can cause diseases for humans and animals (Coenye & Vandamme, 2003). Furthermore, the bacterium is observed to be prevalent in rhizsophere environment for plants (Caballero-Mellado et al., 2007), which may be the reason why the environmental term “rhizosphere” was strongly observed for it as compared to *Acidothermus*. Finally, the presence of the term “sludge” maybe be due to its potential and application for biodegradation (Zhang et al., 2013). Overall, this data support the widespread distribution in plant rhizosphere of these taxa in multiple niches.

#### Marine dataset

*Prochlorococcus*, one of the most abundant organism on the planet (Partensky, Hess & Vaulot, 1999), is typically observed in oligotropic oceans where nutrients availability is poor, in contrast to *Synechococcus* that favours nutrient rich environment (Whitton, 2012). Hence terms such as “ocean” and “brine pool” pointed towards prevalence of *Prochlorococcus* in these environments.

The list of terms for *Synechococcus* includes “reef” and “coast” which are nutrient rich environments compared to oceans. In fact the bacterium has been observed to be present in high abundance at coral reefs especially during summer time (Moriarty, Pollard & Hunt, 1985) as well as coastal regions such as the Portuguese coast (Martins et al., 2005).

Overall, the enhancement provided robust data on taxa-specific distribution in different habitats and highlights the usefulness of this approach for delineating the niches potentially occupied by specific taxon, in this case supporting the known distribution of these abundant marine autotrophs, which drive primary production (Christaki et al., 1999).

## Conclusion

By integrating SEQenv with TaxaSE and extending the functionality through generation of per environment taxa abundance as well as per taxon term abundance data, the improved SEQenv offers unique insights and contributes to the expanding repertoire of next-generation sequence analysis pipelines. This enables the extended pipeline to provide environmental annotations in a variety of contexts.

Furthermore, by directly producing environmental source information for sequences in the dataset, it can greatly help biologists aiming to understand the biogeography of microbes. Given that more and more sequences and genomes are being submitted to the NCBI database, along with associated metadata such as isolation sources, the capabilities of the pipeline would improve in the future.

The system is capable of accurately annotating environmental information to query sequences and enhancement done to SEQenv, which links taxa to environmental keywords, enhances the applicability of this pipeline. This enhancement would play a greater role in helping ecologists understand the diversity patterns present across diverse habitats and will lead to a holistic approach towards ecological projects.

Overall, by understanding the distribution of taxa across niches, next generation sequencing can realize its potential to understand biodiversity and the underlying mechanisms that generate and sustain it. Here, the enhanced SEQenv pipeline integrated with TaxaSE system would serve as an invaluable addition to a biologist’s arsenal of bioinformatics tools.

##  Supplemental Information

10.7717/peerj.3827/supp-1 Supplemental Information 1 Supplementary Material - 1

## Additional Information and Declarations

### DNA Deposition

The following information was supplied regarding the deposition of DNA sequences: Bacterial 16S rDNA sequences—Sugarcane dataset available at:
http://hie-pub.westernsydney.edu.au/6a603c5e-35d6-11e7-b329-525400daae48/.

### Data Availability

The following information was supplied regarding data availability: Seqenv extension and associated data:
http://hie-pub.westernsydney.edu.au/0610b020-39fb-11e7-b55d-525400daae48/. Seqenv pipeline is available at:
https://github.com/xapple/seqenv.
