# Supplementary material for: Extending SEQenv: a taxa-centric approach to environmental annotations of 16S rDNA sequences
Source: PeerJ. 2017 Oct 10;5:e3827. doi: 10.7717/peerj.3827 (PMC5639872; doi:10.7717/peerj.3827)
Supplement: Supplemental Information 1 [file peerj-05-3827-s001.docx]

**Sugarcane Dataset Sampling**

Sugarcane leaf, stalk, root and rhizosphere soil samples were collected by Dr. Kelly Hamonts at Hawkesbury Institute for the Environment, Western Sydney University, Australia, in November 2014 from eight sugarcane fields growing three sugarcane varieties (KQ228, MQ239 and Q240) near Ingham, Queensland, Australia. In each field, 3 stools were randomly selected and samples were collected from 2 plants per stool. Samples were snap-frozen in liquid nitrogen on the field, transported to the laboratory on dry ice and stored at -80C. Frozen sugarcane tissue samples were ground using mortar and pestle and DNA was extracted from the resulting powder using the MoBio PowerPlant DNA extraction kit, following the manufacturer’s instructions. The MoBIO PowerSoil DNA extraction kit was used to extract DNA from the soil samples. Bacterial 16S rRNA amplicon sequencing was performed by the NGS facility at Western Sydney University using Illumina Miseq (2x 301 bp PE) and the 341F/805R primer set.

**SEQenv parameters**

| **SEQenv Parameters** | | |
| --- | --- | --- |
| **Parameter** | **Information** | **Default value used** |
| --min_identity | Minimum identity in similarity search. | 0.97 |
| --min_coverage | Minimum query coverage in similarity search. | 0.97 |
| --proportional | Should we divide the counts of every input sequence by the number of envo terms that were associated to it. | True |
| --search_db | The path to the database to search against. | nt |
| --max_targets | Maximum number of reference matches in similarity search. | 10 |
| --seq_type | Either `nucl` or `prot`. | nucl (nucleotide) |
| --search_algo | Either 'blast' or 'usearch'. | blast |
| --e_value | Minimum e-value in similarity search. | 0.0001 |

**List of tools developed for SEQenv extension**

| **Tool** | **Description** |
| --- | --- |
| seqenv-selector.jar | This tool selects sequences based on taxonomic annotation and abundance data present in TaxaSE system results. |
| seqenv-abd.jar | This tool aggregates the results on SEQenv pipeline on a per-term basis and generates a list of most abundant environmental terms. |
| seqenv-cloud-gen.jar | As the current version of SEQenv does not provide word cloud generation functionality, this tool was developed to illustrate the pipeline results. |
| seqenv-rev.jar | This tool represents the extension to the SEQenv pipeline. By using the TaxaSE annotation information and SEQenv results, this tool is able to generate both per term taxa abundance and per taxa term abundance results. |
